# Supplementary material for: Local axonal morphology guides the topography of interneuron myelination in mouse and human neocortex
Source: eLife. 2019 Nov 19;8:e48615. doi: 10.7554/eLife.48615 (PMC6927753; doi:10.7554/eLife.48615)
Supplement: Supplementary file 1. [file elife-48615-supp1.docx]

**Supplementary File 1.** Electrophysiological properties of PV::Ai14 cells

|  | ***Pvalb::cre,*Ai14** | *n = 18* |
| --- | --- | --- |
|  | average | *s.e.* |
| Ri (MΩ) | 147.48 | *12.57* |
| RMP (mV) | -71.05 | *1.32* |
| AP Threshold (mV) | -36.44 | *2.98* |
| AP Amplitude (mV) | 77.18 | *2.57* |
| AP Frequency (Hz) | 128.30 | *5.60* |
| AP Half-width (ms) | 0.39 | *0.02* |
| AP Rise time (ms) | 0.19 | *0.01* |
| fAHP Amplitude (mV) | -19.84 | *0.84* |

Abbreviations: Ri Input resistance, RMP resting membrane potential, AP action potential, fAHP fast afterhyperpolarization. AP frequency determined with 500ms square-wave current pulse at +400 pA.
