# Supplementary material for: Local axonal morphology guides the topography of interneuron myelination in mouse and human neocortex
Source: eLife. 2019 Nov 19;8:e48615. doi: 10.7554/eLife.48615 (PMC6927753; doi:10.7554/eLife.48615)
Supplement: Supplementary file 2. [file elife-48615-supp2.docx]

**Supplementary File 2.** Electrophysiological properties of SOM::Ai14 cells

|  | ***Sst::cre,*Ai14** | *n =* 13 |
| --- | --- | --- |
|  | average | *s.e.* |
| Ri (MΩ) | 268.05 | *23.05* |
| Sag (mV) | -1.68 | *0.29* |
| RMP (mV) | -67.76 | *1.88* |
| AP Threshold (mV) | -45.71 | *2.80* |
| AP Amplitude (mV) | 88.65 | *2.84* |
| AP Frequency (Hz) | 64.4 | *2.10* |
| AP Half-width (ms) | 0.71 | *0.03* |
| AP Rise time (ms) | 0.25 | *0.01* |
| fAHP Amplitude (mV) | -11.36 | *1.40* |

Abbreviations: Ri Input resistance, RMP resting membrane potential, AP action potential, fAHP fast afterhyperpolarization. AP frequency determined with 500ms square-wave current pulse at +400 pA.
