# Supplementary material for: Local axonal morphology guides the topography of interneuron myelination in mouse and human neocortex
Source: eLife. 2019 Nov 19;8:e48615. doi: 10.7554/eLife.48615 (PMC6927753; doi:10.7554/eLife.48615)
Supplement: Supplementary file 3. [file elife-48615-supp3.docx]

**Supplementary File 3.** Electrophysiological properties of human fast-spiking interneurons

|  | ***Human FS Interneurons*** | *n = 4* |
| --- | --- | --- |
|  | average | *s.e.* |
| Ri (MΩ) | 144.58 | *12.34* |
| RMP (mV) | -64.27 | *1.92* |
| AP Threshold (mV) | -42.31 | *1.28* |
| AP Amplitude (mV) | 88.82 | *1.20* |
| AP Frequency (Hz) | 164.00 | *5.73* |
| AP Half-width (ms) | 0.33 | *0.02* |
| AP Rise time (ms) | 0.21 | *0.01* |
| fAHP Amplitude (mV) | -18.81 | *1.09* |

Abbreviations: Ri Input resistance, RMP resting membrane potential, AP action potential, fAHP fast afterhyperpolarization. AP frequency determined with 500ms square-wave current pulse at +400 pA. FS fast-spiking
